# Therapeutic Software Interventions in Japan: A Maturity Map Derived from PubMed Records (2015-2026)

**DOI:** 10.31662/jmaj.2026-0080

**Published:** 2026-07-24

**Authors:** Shota Miyakuni

**Affiliations:** 1 medsuke Inc., Tokyo, Japan

**Keywords:** digital therapeutics, software as a medical device, scoping review, Japan, implementation, economic evaluation

## Introduction

Therapeutic software interventions (including digital therapeutics [DTx] and software as a medical device [SaMD]) deliver evidence-based therapy through software ^(1), (2)^. However, PubMed-indexed publications reporting studies conducted in Japan remain dispersed across disease areas, study designs, and outcome types. To provide a compact and reproducible snapshot of the distribution of published evidence and remaining evidence gaps, we mapped PubMed-indexed publications (2015-2026) along two operational axes: (i) evaluation rigor (feasibility/pilot, non-randomized comparative, randomized controlled trial; protocol-only publications excluded) and (ii) clinical proximity of the primary outcome (from engagement proxies to hard clinical end points).

## Methods

We conducted a PubMed-based scoping review ^(3), (4), (5)^. The unit of analysis was each publication; when multiple publications arose from the same study, each eligible publication was included. The maturity map was intended as a descriptive evidence-mapping framework rather than a validated maturity scale. Its two axes were selected pragmatically to summarize visible PubMed-indexed clinical evidence, not to fully capture internal validity, intervention complexity, regulatory status, implementation readiness, or real-world adoption. AI-assisted tools/technologies (ChatGPT; OpenAI) were used for language editing and internal consistency checks; study selection, extraction, classification, and interpretation were led by the author. We searched PubMed (January 1, 2015 to February 1, 2026) using three pre-defined queries targeting SaMD/DTx-related terms and mobile/web-based therapeutic interventions; full strategies, exclusions, record accounting, and known-item check details are presented in Supplementary Appendix S1. We excluded review articles, editorial/comment/letter/news publication types, and animal-only studies. Records were restricted to those with abstracts, merged, and de-duplicated by PMID. Screening was based primarily on titles/abstracts using a PCC framework: clinical/treatment populations, software-delivered therapeutic interventions, and Japan execution. Full texts were consulted when required to adjudicate eligibility (e.g., Japan execution) or to classify primary outcomes. Protocol-only publications without outcome data were excluded from the final dataset. We also ran a pre-specified known-item check (intervention/product names) to detect prominent Japan-originated therapeutic software reports missed by Q1-Q3. The author takes full responsibility for the content, accuracy, and integrity of the manuscript.

For each included publication, we charted disease area, software modality, study design, primary outcome, and whether the abstract/full text described implementation-relevant features (implementation outcomes, economic evaluation, and human support) ^(6)^. Evaluation rigor was classified as F (feasibility/pilot), N (non-randomized comparative or observational/pre–post with outcomes), and R (randomized controlled trials [RCTs], including cluster RCTs). Protocol-only publications were excluded in the dataset by design. Decision-analytic modeling studies (e.g., cost-effectiveness simulations without empirical clinical outcome data) were not included. Because this is a publication-level mapping, rigor was assigned on the basis of the analytic contrast reported in each publication; secondary analyses without randomized between-group inference were categorized as N even if originating from an RCT. Clinical proximity of the primary outcome was classified as 1 (engagement/behavioral proxy), 2 (symptom scores/patient-reported outcomes [PROs]), 3 (disease indicators/biomarkers), 4 (health care utilization), and 5 (hard clinical end points). We summarized distributions descriptively and visualized counts on a maturity map (X = rigor; Y = proximity). To evaluate the influence of publication-level counting, we also assigned each publication to a study/trial identifier and conducted a study/trial-level sensitivity analysis. Secondary or post hoc publications clearly derived from the same underlying trial or study program were retained in the publication-level dataset but were not counted as representative publications in this sensitivity analysis. Minimal methodological characteristics, including analysis type and key design features, were summarized descriptively without conducting a formal risk-of-bias assessment or excluding publications on this basis (Supplementary Table S2).

### Results

Study selection is summarized in Figure 1. By evaluation rigor, RCTs accounted for 16/24 (66.7%), non-randomized comparative/observational designs for 5/24 (20.8%), and feasibility/pilot studies for 3/24 (12.5%). Primary outcomes were distributed across proximity level 1 (n = 9), 2 (n = 7), and 3 (n = 8), with no level 4-5 primary outcomes. Implementation-related reporting was present in 6/24 (25.0%), human support components in 7/24 (29.2%), and no within-trial economic evaluation was reported (0/24); decision-analytic cost-effectiveness models exist but were outside the scope ^(7)^. In the study/trial-level sensitivity analysis, three secondary or post hoc publications were grouped with their corresponding primary trial reports, yielding 21 units; the overall pattern remained similar (R, 15/21; N, 3/21; F, 3/21), with outcomes still concentrated in levels 1-3. The maturity map showed RCTs concentrated in levels 1-3, particularly in the RCT × symptom/PRO cell (7 publications; Figure 2). Disease areas were largely cardiometabolic/cardiovascular (11/24) or mental health/addiction/sleep (11/24), with essential hypertension the most frequent single indication (6/24).

**Figure 1.**
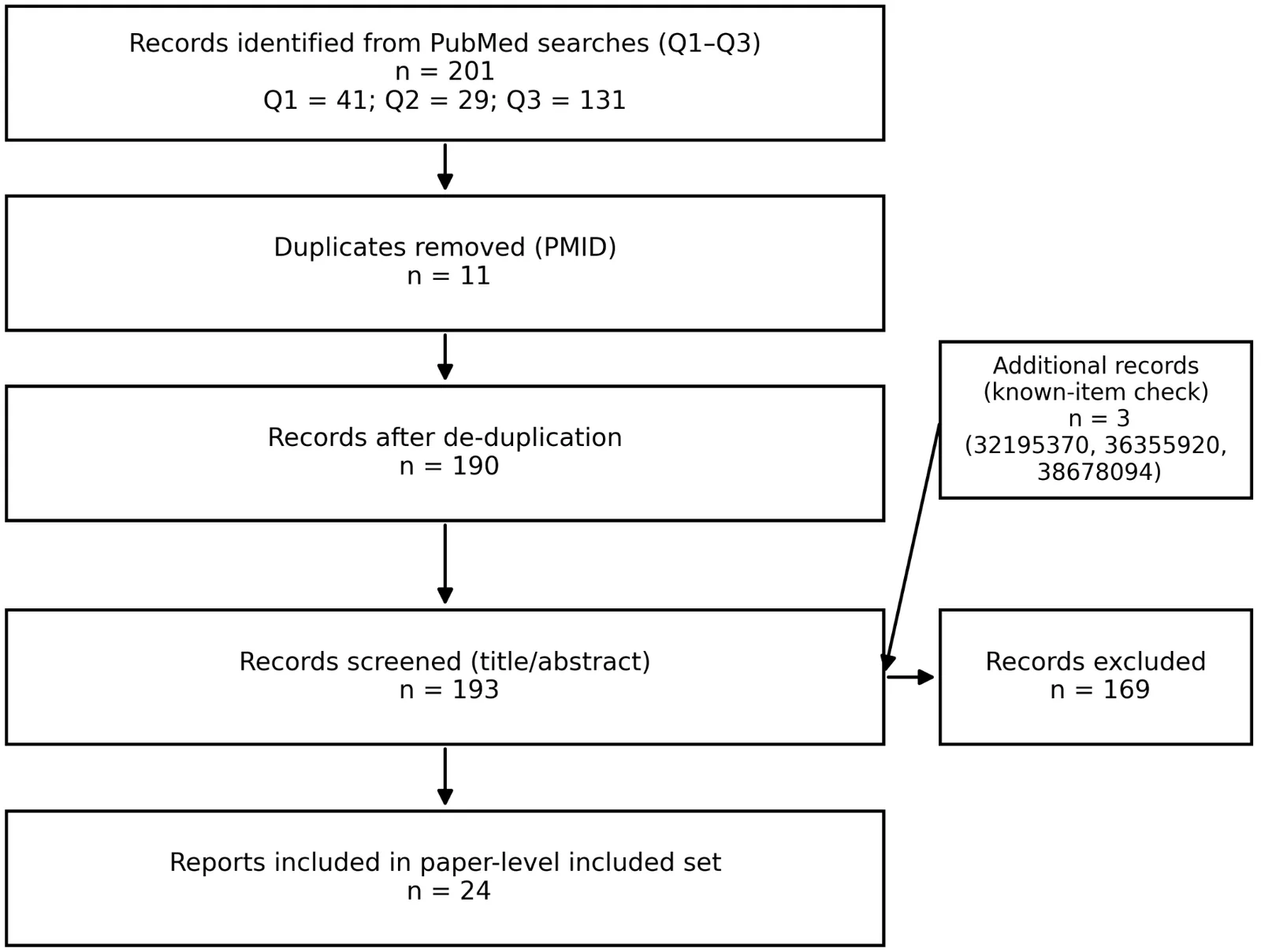
Study selection flow (PubMed Q1-Q3 plus known-item check). Records were retrieved using Q1-Q3; duplicates were removed by PMID; three additional records were added through a known-item check; titles/abstracts were screened, yielding 24 publications.

**Figure 2.**
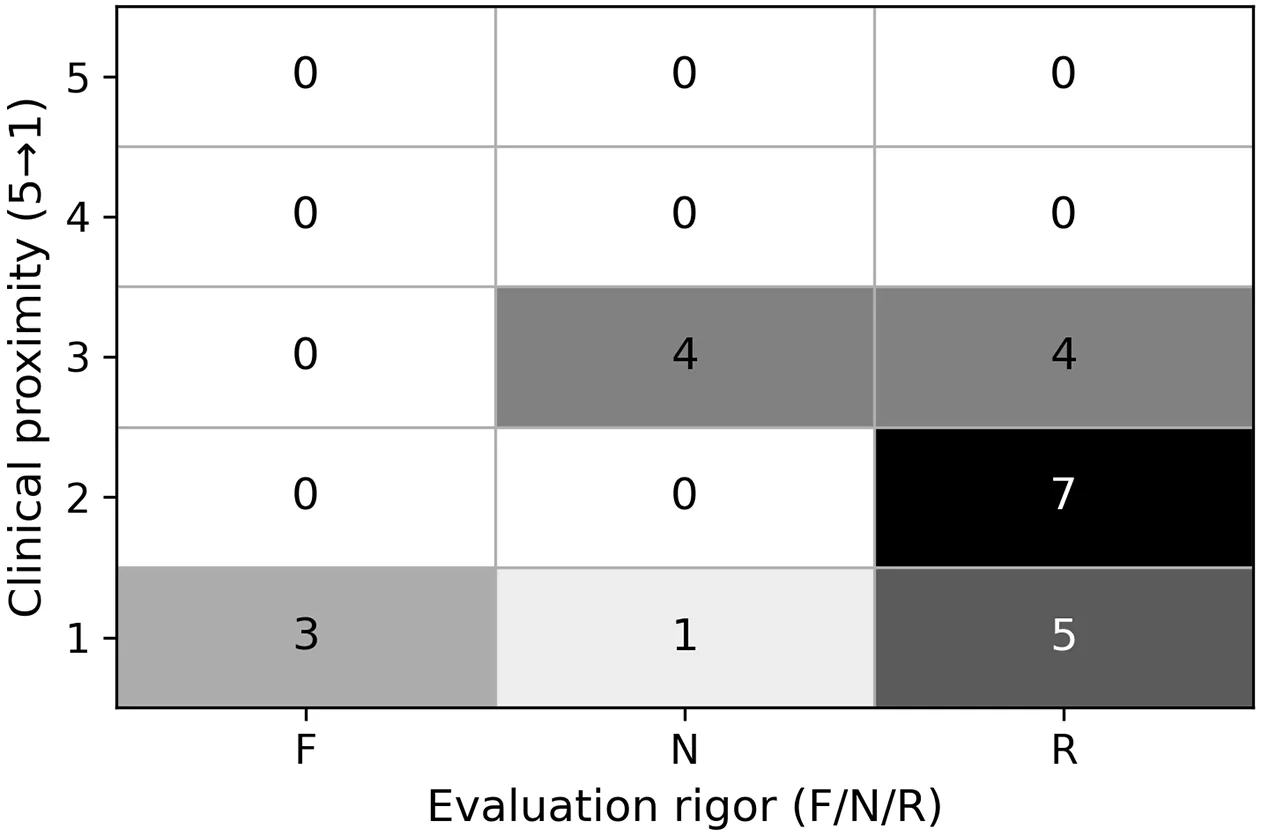
Maturity map counts (n = 24; included publications). Cells indicate counts of publications per evaluation rigor × clinical proximity combination. Evaluation rigor: F, feasibility/pilot; N, non-randomized, including pre–post studies and secondary analyses without randomized between-group inference; R, randomized controlled trials. Clinical proximity: levels 1-5, as defined in the Methods. No included publication used clinical proximity levels 4-5 (health care utilization or hard clinical end points) as the primary outcome.

## Discussion

Across PubMed-indexed therapeutic software publications reporting studies conducted in Japan (2015-2026), two-thirds reported randomized evaluation, but primary outcomes clustered at proximal levels (engagement/behavioral proxies, symptoms/PROs, biomarkers), with no included publication using health care utilization or hard clinical end points as the primary outcome. This predominance of RCT publications should not be interpreted as indicating uniformly high internal validity or implementation readiness because evaluation rigor in this map was based on study design category and analytic contrast rather than a formal risk-of-bias assessment. Implementation-oriented reporting was limited, and within-trial economic evaluation was absent. These patterns likely reflect the time horizon and mechanism of many DTx (behavior change and care-process optimization): intermediate outcomes, including symptom/PRO changes and care-process measures such as clinical inertia, may be meaningful proximal targets that precede downstream utilization and events.

Rather than implying that fields lacking level 4-5 outcomes are “immature,” the maturity map should be interpreted as a descriptive snapshot of evidence distribution under operational definitions of rigor and outcome proximity. For several indications, validated symptom/PRO improvement may represent clinically meaningful benefit. Nonetheless, the scarcity of implementation and economic reporting may indicate a gap between evidence typically generated in academic trials and evidence often needed for real-world adoption and reimbursement. Economic evidence may also be generated through decision-analytic models or non-public materials outside journal articles. Future work could link proximal end points to downstream outcomes through longer follow-up, pragmatic trials, and real-world data, while incorporating hybrid effectiveness–implementation designs, pre-specified implementation outcomes, payer-relevant end points, and parallel economic evaluation according to each intervention’s mechanism and intended use.

Limitations include reliance on PubMed and abstract availability, which may preferentially capture internationally published and well-resourced studies while underrepresenting early-stage development work, negative results, domestic-only publications, and industry-generated evidence. Therefore, these findings should be interpreted as a map of internationally indexed published evidence rather than a comprehensive assessment of all therapeutic software development or implementation activity in Japan. Restrictive Q3 NOT terms improved specificity for patient-treatment contexts but may have excluded relevant records; this was partially mitigated through a known-item check. Screening and coding were performed by a single reviewer without duplicate assessment. The primary analysis remained publication-level, and although the study/trial-level sensitivity analysis reduced double counting of clearly linked secondary or post hoc publications, grouping decisions relied on reported information. Rigor/proximity classifications may be imperfect, and the methodological characterization was descriptive rather than a formal appraisal of internal validity. The axes also do not capture intervention complexity, regulatory status, or implementation readiness.

## Conclusion

Among PubMed-indexed therapeutic software publications reporting studies conducted in Japan (2015-2026), RCT publications accounted for two-thirds of included publications (16/24), although the absolute number was limited and should not be equated with uniformly high internal validity. Primary outcomes rarely extended to health care utilization or hard clinical end points, and implementation/economic reporting remained sparse.

## Article Information

### Author Contributions

Shota Miyakuni undertook conceptualization; data curation; formal analysis; methods; visualization; writing—original draft; and writing—review and editing.

### Conflicts of Interest

Shota Miyakuni is affiliated with medsuke Inc. and has had a professional relationship with asken Inc. within the past 36 months; asken Inc. had no role in this study. The author declares no other conflicts of interest.

### Ethics Approval/IRB

Not applicable. This study analyzed publicly available published literature and did not involve human participants or identifiable personal data.

### Data Availability

All included publications, extracted variables, study/trial-level grouping, and methodological characterization are presented in Supplementary Tables S1 and S2.

## Supplement

Supplementary Material
