# Supplementary material for: Therapeutic Software Interventions in Japan: A Maturity Map Derived from PubMed Records (2015-2026)
Source: JMA J. 2026 Jul 24;9(5):1389–92. doi: 10.31662/jmaj.2026-0080 (PMC13639558; doi:10.31662/jmaj.2026-0080)
Supplement: Supplementary file 1 [file 2433-3298-9-5-1389-s001.pdf]

Supplementary Appendix S1. PubMed search strategy (Q1–Q3) and known-item check

Database: PubMed

Time window: January 1, 2015 to February 1, 2026 (dp)

Last searched on: 2026-02-14

Export: Records were exported in .nbib format and merged; duplicates were removed by PMID.

Core limits/exclusions applied across queries: abstract available; exclude review articles; exclude editorial/comment/letter/news publication types; and exclude animal-only studies.

Q1 (high precision: DTx / therapeutic app terms)

Hits at execution: 41

```
(
  "digital therapeutics"[tiab]
  OR "digital therapeutic"[tiab]
```

OR "therapeutic app"[tiab]

OR "therapeutic apps"[tiab]

)

AND (Japan[ad] OR Japan[tiab])

AND ("2015/01/01"[dp] : "2026/02/01"[dp])

AND "hasabstract"[text]

NOT review[pt]

NOT (animals[mh] NOT humans[mh])

NOT (editorial[pt] OR comment[pt] OR letter[pt] OR news[pt])

Q2 (complementary: SaMD/DTx terms + evaluation signals)

Hits at execution: 29

(

SaMD[tiab]

OR "software as a medical device"[tiab]

OR "therapeutic software"[tiab]

OR DTx[tiab]

)

AND (trial[tiab] OR randomized[tiab] OR randomised[tiab] OR protocol[tiab] OR feasibility[tiab] OR pilot[tiab] OR controlled[tiab])

AND (Japan[ad] OR Japan[tiab])

AND ("2015/01/01"[dp] : "2026/02/01"[dp])

AND "hasabstract"[text]

NOT review[pt]

NOT (animals[mh] NOT humans[mh])

NOT (editorial[pt] OR comment[pt] OR letter[pt] OR news[pt])

Q3 (complementary: mobile/web interventions + RCT[pt] + treatment terms; exclude workplace-focused terms)

Hits at execution: 131

```
(
  (
    "smartphone app"[tiab]
    OR "mobile app"[tiab]
    OR "smartphone application"[tiab]
    OR "mobile application"[tiab]
```

OR mhealth[tiab]

OR "mobile health"[tiab]

OR "web-based"[tiab]

OR "internet-based"[tiab]

)

AND "randomized controlled trial"[pt]

AND (intervention*[tiab] OR treatment*[tiab] OR therap*[tiab] OR rehabilitation[tiab] OR "self-management"[tiab])

AND (Japan[ad] OR Japan[tiab] OR Japanese[tiab])

)

AND ("2015/01/01"[dp] : "2026/02/01"[dp])

AND "hasabstract"[text]

NOT review[pt]

NOT (animals[mh] NOT humans[mh])

NOT (workplace[tiab] OR employee*[tiab] OR occupational[tiab] OR worksite[tiab] OR "health promotion"[tiab] OR healthy[tiab])

Record accounting (search run)

Q1–Q3 yielded 201 records in total. After de-duplication by PMID (11 duplicates removed), 190 unique records remained. A pre-specified known-item check added 3 additional records, resulting in 193 screened records.

Known-item check keywords (used to minimize false negatives; examples): CureApp, CASC, HERB, HERB-DH1, SDT-001, SUSMED, Yukumi, DialBetesPlus.

Known-item check added records (PMIDs): 32195370, 36355920, 38678094.

Supplementary Table S1. Included publications and extracted variables

PMID	year	journal	title	doi	disease_area_primary	software_mode	evaluation_rigor	clinical_proximity	implementation_flag	human_support_flag	economics_flag
29101095	2017	Journal of medical Internet research	Smartphone Cognitive Behavioral Therapy as an Adjunct to Pharmacotherapy for Refractory Depression: Randomized Controlled Trial.	10.2196/jmir.8602	Major depressive disorder	App	R	2	0	0	0
30249583	2018	Journal of medical Internet research	Web-Based Cognitive Behavioral Therapy Blended With Face-to-Face Sessions for Major Depression: Randomized Controlled Trial.	10.2196/10743	Major depressive disorder	Web	R	2	0	1	0
30777848	2019	JMIR mHealth and uHealth	Impact of a Novel Smartphone App (CureApp Smoking Cessation) on Nicotine Dependence: Prospective Single-Arm Interventional Pilot Study.	10.2196/12694	Tobacco use disorder (smoking cessation)	Device-tied	F	1	1	1	0

PMID	year	journal	title	doi	disease_area_primary	software_mode	evaluation_rigor	clinical_proximity	implementation_flag	human_support_flag	economics_flag
32087837	2020	Journal of substance abuse treatment	Effect of a web-based relapse prevention program on abstinence among Japanese drug users: A pilot randomized controlled trial.	10.1016/j.jsat.2019.12.001	Substance use disorder (drug relapse prevention)	Web	R	1	1	0	0
32195370	2020	NPJ Digital Medicine	A randomized controlled trial of a smoking cessation smartphone application with a carbon monoxide checker	10.1038/s41746-020-0243-5	Tobacco use disorder (smoking cessation)	Device-tied	R	1	0	1	0
32201165	2020	Clinical breast cancer	Effectiveness of a Smartphone Application as a Support Tool for Patients Undergoing Breast Cancer Chemotherapy: A Randomized Controlled Trial.	10.1016/j.clbc.2020.01.004	Breast cancer (supportive care during chemotherapy)	App	R	2	0	0	0
33484628	2021	Journal of clinical hypertension	Digital therapeutics for essential hypertension using a smartphone application: A	10.1111/jch.14191	Essential hypertension	Mixed	R	3	0	0	0

PMID	year	journal	title	doi	disease_area_primary	software_mode	evaluation_rigor	clinical_proximity	implementation_flag	human_support_flag	economics_flag
		(Greenwich, Conn.)	randomized, open-label, multicenter pilot study.								
34455443	2021	European heart journal	Efficacy of a digital therapeutics system in the management of essential hypertension: the HERB-DH1 pivotal trial.	10.1093/eurheartj/ehab559	Essential hypertension	Mixed	R	3	0	0	0
35689457	2022	Neuropsychopharmacology reports	Effects of a web-based relapse prevention program on abstinence: Secondary subgroup analysis of a pilot randomized controlled trial.	10.1002/npr2.12272	Substance use disorder (drug relapse prevention)	Web	R	1	0	0	0
36355920	2023	Sleep	Effect of smartphone-based cognitive behavioral therapy app on insomnia: a randomized, double-blind study	10.1093/sleep/zsac270	Insomnia disorder	App	R	2	0	0	0
36656974	2023	The American journal of	Impact of a Novel Digital Therapeutics System on Nonalcoholic	10.14309/ajg.00	Nonalcoholic steatohepa	App	N	3	1	1	0

PMID	year	journal	title	doi	disease_area_primary	software_mode	evaluation_rigor	clinical_proximity	implementation_flag	human_support_flag	economics_flag
		gastroenterology	Steatohepatitis: The NASH App Clinical Trial.	00000000002143	titis (NASH)						
36899182	2023	Hypertension research : official journal of the Japanese Society of Hypertension	Home blood pressure-lowering effect of digital therapeutics in hypertension: impact of body weight and salt intake.	10.1038/s41440-023-01245-7	Essential hypertension	Mixed	N	3	0	0	0
37717116	2024	Hypertension research : official journal of the Japanese Society of Hypertension	Daily self-reported behavioural efficacy records on hypertension digital therapeutics as digital metrics associated with the reduction in morning home blood pressure: post-hoc analysis of HERB-DH1 trial.	10.1038/s41440-023-01434-4	Essential hypertension	Mixed	N	3	0	0	0
37800348	2023	Acta dermato-venereologica	Monitoring Sleep and Scratch Improves Quality of Life in Patients with Atopic Dermatitis.	10.2340/actadv.v103.11922	Atopic dermatitis	Device-tied	R	2	0	0	0

PMID	year	journal	title	doi	disease_area_primary	software_mode	evaluation_rigor	clinical_proximity	implementation_flag	human_support_flag	economics_flag
38073868	2023	Circulation reports	A Heart Failure Smartphone Application That Nudges Patients/Physicians Toward Optimal Medical Therapy - Development and Usability Study.	10.1253/circrep.CR-23-0088	Chronic heart failure	Mixed	F	1	1	1	0
38468404	2024	Psychiatry and clinical neurosciences	Optimizing smartphone psychotherapy for depressive symptoms in patients with cancer: Multiphase optimization strategy using a decentralized multicenter randomized clinical trial (J-SUPPORT 2001 Study).	10.1111/pcn.13657	Depressive symptoms (cancer patients)	App	R	2	0	0	0
38678094	2024	NPJ Digital Medicine	Effectiveness of DialBetesPlus, a self-management support system for diabetic kidney disease: Randomized controlled trial	10.1038/s41746-024-01114-8	Type 2 diabetes with chronic kidney disease	App	R	3	0	0	0

PMID	year	journal	title	doi	disease_area_primary	software_mode	evaluation_rigor	clinical_proximity	implementation_flag	human_support_flag	economics_flag
39024702	2024	General hospital psychiatry	A smartphone app-based intervention combined with face-to-face sessions for alcohol dependence at internal medicine clinics: A randomized controlled trial.	10.1016/j.genhosppsych.2024.07.003	Alcohol use disorder	App	R	1	0	1	0
39255478	2024	Journal of medical Internet research	Efficacy of Save Medical Corporation (SMC)-01, a Smartphone App Designed to Support Type 2 Diabetes Self-Management Based on Established Guidelines: Randomized Controlled Trial.	10.2196/53740	Type 2 diabetes	App	R	3	0	0	0
39394509	2025	Hypertension research : official journal of the Japanese Society of Hypertension	Digital therapeutic for hypertension improves physician-patient communication and clinical inertia: a survey of physicians who implemented CureApp HT in clinical practice.	10.1038/s41440-024-01899-x	Essential hypertension	Mixed	N	1	1	0	0

PMID	year	journal	title	doi	disease_area_primary	software_mode	evaluation_rigor	clinical_proximity	implementation_flag	human_support_flag	economics_flag
40153547	2025	JMIR formative research	Personalized mHealth Intervention (StepAdd) for Increasing Physical Activity in Japanese Patients With Type 2 Diabetes: Secondary Analysis of Social Cognitive Theory Measurements of a Single-Arm Pilot Study.	10.2196/60221	Type 2 diabetes	App	F	1	1	0	0
40317565	2025	Psychiatry and clinical neurosciences	Efficacy and safety of SDT-001, a dual-task digital device, in managing attention-deficit/hyperactivity disorder symptoms in children and adolescents: a phase 3, randomized, standard treatment-controlled study.	10.1111/pcn.13833	Attention-deficit/hyperactivity disorder (ADHD)	Device-tied	R	2	0	0	0
40719411	2025	Psychiatry and clinical neurosciences	Efficacy and safety of a digital therapeutic for alcohol dependence: A multicenter, open-label,	10.1111/pcn.13874	Alcohol use disorder	App	R	1	0	1	0

PMID	year	journal	title	doi	disease_area_primary	software_mode	evaluation_rigor	clinical_proximity	implementation_flag	human_support_flag	economics_flag
			randomized controlled trial.								
41178557	2026	Hypertension (Dallas, Tex. : 1979)	Efficacy Determinants of Hypertension Digital Therapeutics: The B-INDEX Study.	10.1161/HYPERTENSIONAHA.125.25028	Essential hypertension	Mixed	N	3	0	0	0

Note: Software mode reflects the primary digital delivery mode. Adjunct face-to-face or clinician support was captured separately by the human_support_flag.

Supplementary Table S2. Study/program-level grouping and minimal methodological characterization

PMID	study_or_trial_id	analysis_type	representative_for_sensitivity_analysis	methodological_characterization	grouping_note
29101095	MDD-CBT-app-2017	Primary RCT	Yes	RCT; app adjunct to pharmacotherapy	Independent study
30249583	MDD-CBT-blended-2018	Primary RCT	Yes	RCT; blended web-based CBT and face-to-face sessions	Independent study
30777848	CureApp-smoking-pilot	Single-arm pilot	Yes	Prospective single-arm pilot	Earlier-stage smoking cessation app study
32087837	Drug-relapse-web-RCT	Pilot RCT / primary report	Yes	Pilot RCT	Representative for related secondary subgroup analysis
32195370	CureApp-smoking-RCT	Primary RCT	Yes	RCT; app with carbon monoxide checker	Independent trial-level unit
32201165	Breast-cancer-chemo-app	Primary RCT	Yes	RCT	Independent study
33484628	HERB-HT-pilot	Pilot RCT / primary report	Yes	Randomized open-label multicenter pilot	Separate from pivotal HERB-DH1 trial
34455443	HERB-DH1	Primary pivotal RCT	Yes	Pivotal RCT	Representative for HERB-DH1-related secondary/post-hoc analyses
35689457	Drug-relapse-web-RCT	Secondary subgroup analysis	No	Secondary subgroup analysis of pilot RCT; randomized contrast retained	Grouped with PMID 32087837
36355920	Insomnia-CBT-app	Primary RCT	Yes	Randomized double-blind study	Independent study
36656974	NASH-app-trial	Primary non-randomized trial	Yes	Non-randomized interventional study	Independent study
36899182	HERB-DH1	Secondary analysis	No	Secondary analysis using HERB-DH1 data; non-randomized analytic contrast	Grouped with PMID 34455443
37717116	HERB-DH1	Post-hoc analysis	No	Post-hoc analysis of HERB-DH1 trial; non-randomized analytic contrast	Grouped with PMID 34455443

PMID	study_or_trial_id	analysis_type	representative_for_sensitivity_analysis	methodological_characterization	grouping_note
37800348	AD-sleep-scratch-device	Primary RCT	Yes	RCT; device-tied monitoring intervention	Independent study
38073868	HF-app-usability	Development/usability study	Yes	Development and usability / feasibility study of patient–physician-facing heart failure app	Independent feasibility-level unit
38468404	J-SUPPORT-2001	Randomized optimization trial	Yes	Decentralized multicenter randomized clinical trial	Independent study
38678094	DialBetesPlus-DKD	Primary RCT	Yes	RCT	Independent study
39024702	AUD-app-face-to-face	Primary RCT	Yes	RCT; app plus face-to-face sessions	Independent study
39255478	SMC-01-T2D	Primary RCT	Yes	RCT	Independent study
39394509	CureApp-HT-implementation-survey	Implementation survey	Yes	Physician survey / observational implementation study	Real-world implementation-related publication
40153547	StepAdd-T2D	Secondary analysis of single-arm pilot	Yes	Secondary analysis of single-arm pilot	Treated as separate pilot program
40317565	SDT-001-ADHD	Phase 3 RCT	Yes	Phase 3 randomized controlled study	Independent study
40719411	AUD-DTx-RCT	Primary RCT	Yes	Multicenter open-label RCT	Independent study
41178557	B-INDEX-HT	Multicenter intervention / determinant analysis	Yes	Multicenter non-randomized intervention/determinant analysis	Treated as independent from HERB-DH1

Abbreviations: RCT, randomized controlled trial; CBT, cognitive behavioral therapy; AD, atopic dermatitis; ADHD, attention-deficit/hyperactivity disorder; AUD, alcohol use disorder. Publications marked “No” in the representative column were retained in the publication-level dataset but excluded from the study/program-level sensitivity analysis to reduce double counting of secondary or post-hoc analyses derived from the same underlying trial or clinical program. This descriptive characterization was not a formal risk-of-bias assessment and was not used to exclude publications.
